# Prevalencia de leptospirosis en perros de trabajo vacunados y en población humana con riesgo ocupacional

**DOI:** 10.7705/biomedica.5009

**Published:** 2020-08-20

**Authors:** César A. Murcia, Miryam Astudillo, Marlyn H. Romero

**Affiliations:** 1 Maestría en Ciencias Veterinarias, Universidad de Caldas, Manizales, Colombia Universidad de Caldas Universidad de Caldas Manizales Colombia; 2 Escuela de Ciencias Básicas, Universidad del Valle, Cali, Colombia Universidad del Valle Universidad del Valle Cali Colombia; 3 Grupo CIENVET, Departamento Salud Animal, Facultad de Ciencias Agropecuarias, Universidad de Caldas, Manizales, Colombia Universidad de Caldas Departamento Salud Animal Facultad de Ciencias Agropecuarias Universidad de Caldas Manizales Colombia

**Keywords:** Leptospira, estudios seroepidemiológicos, pruebas de aglutinación, factores de riesgo, perros, Leptospira, seroepidemiologic studies, agglutination tests, risk factors, dogs

## Abstract

**Introducción.:**

Los perros de trabajo pueden infectarse con diversas serovariedades de *Leptospira* que se mantienen en sus túbulos renales e intersticios por mucho tiempo, por lo que se convierten en portadores y fuentes de infección para otros huéspedes.

**Objetivo.:**

Establecer la prevalencia de *Leptospira* spp. en perros de trabajo vacunados y en población humana con riesgo ocupacional de seis unidades policiales en Colombia.

**Materiales y métodos.:**

Mediante tres instrumentos estructurados, se evaluaron 92 perros de trabajo con inmunización previa contra *Leptospira* spp. (65 machos y 27 hembras) y 69 personas de seis unidades policiales de los municipios de Manizales, Pereira, Armenia, Ibagué, Tuluá y Cali. Se obtuvieron muestras sanguíneas de las personas y de los perros, las cuales se evaluaron mediante la prueba de microaglutinación (*Microscopic Agglutination Test*, MAT) en 24 serogrupos. Se hizo un examen clínico de los perros y se obtuvieron muestras de orina para urocultivo.

**Resultados.:**

La seroprevalencia de leptospirosis en las personas fue de 2,9 % (n=2) y en los perros de 57,61 % (n=53). Los serogrupos más prevalentes en los perros fueron *Leptospira canicola* y *L. panama.* El urocultivo fue positivo en 58,7 % (54/92) de las muestras y se encontró asociación estadísticamente significativa entre la edad de los perros (≥10 años; p=0,043) y la ubicación de la unidad policial (p=0,016).

**Conclusión.:**

Las características epidemiológicas de la leptospirosis en los perros sugieren una presentación endémica de la infección. Se requieren algoritmos diagnósticos sensibles y específicos para investigar la leptospirosis canina y diferenciar los anticuerpos vacunales de la infección natural.

## Abstract (en)

**Introduction:**

Working dogs have been identified as a risk group for developing leptospirosis because they can be infected by *Leptospira* spp., which can be kept in the renal tubules and interstices for a long time, making them carriers and sources of infection for other hosts, including humans.

**Objective:**

To establish the prevalence of *Leptospira* spp. in vaccinated working dogs and in the occupationally exposed population in six police units in Colombia.

**Materials and methods:**

A total of 92 vaccinated dogs (65 males and 27 females) and 69 people from six police units in the municipalities of Manizales, Pereira, Armenia, Ibagué, Tuluá, and Cali were tested. Three structured instruments were applied and blood samples were obtained from people and dogs, which were processed with the microagglutination test (MAT) in 24 serogroups. A clinical examination of the dogs was performed and urine samples were obtained for urine cultures.

**Results:**

The seroprevalence of human leptospirosis was 2.9% (n=2) and in dogs, it was 57.61% (n=53). Among the dogs, serogroups *L. canicola and L. pana*ma were the most prevalent. Urine cultures were positive in 58.7% (54/92) of the samples. A statistically significant association was found between the age of the dogs (≥10 years; p=0.043) and the location of the police unit (p=0.016) with the urine culture.

**Conclusion:**

The epidemiological characteristics of leptospirosis in dogs suggest an endemic presentation of the infection. There is an urgent need to improve current diagnostic methods to investigate canine leptospirosis and differentiate between vaccine and natural infection antibodies.

La leptospirosis es una zoonosis de distribución mundial ocasionada por una bacteria del género *Leptospira*; afecta al hombre y a una gran variedad de especies animales, entre ellas, los perros ^1^^,^^2^. La enfermedad se transmite por contacto directo con orina u otros fluidos corporales infectados, o indirectamente después del contacto con agua o suelos contaminados ^3^. Los perros juegan un papel importante en la transmisión de la leptospirosis a los seres humanos y a otros animales domésticos ^4^.

Los perros de trabajo son un grupo de animales entrenados para desarrollar actividades de asistencia humana, por ejemplo, servicio de guardia, ayuda terapéutica, soporte a operaciones de rescate, guías y cuidadores de personas invidentes o discapacitadas, y guías policiales, así como en la detección de estupefacientes y explosivos, entre otras ^1^. Los cuidadores y manejadores de estos animales tienen una interacción muy estrecha con ellos, lo que exige cuidados de salud y medidas preventivas apropiadas. Los perros de trabajo son un grupo de riesgo para desarrollar leptospirosis ^1^.

Estos perros son inmunizados rutinariamente contra la leptospirosis, pero varios autores han descrito fallas en la capacidad del producto biológico para generar inmunidad y problemas asociados con la protección incompleta que confieren las vacunas bacterianas comerciales, aspecto que aumenta la probabilidad de transmisión de la leptospirosis por los perros a los humanos y otros animales propensos ^3^^,^^5^. Por otra parte, la prueba de microaglutinación (*Microscopic Agglutination Test*, MAT) se utiliza ampliamente en los estudios epidemiológicos y la confirmación clínica de la leptospirosis canina, pero esta no discrimina los anticuerpos aglutinantes relacionados con la infección de los que se desarrollan como producto de la vacunación, aspecto que dificulta la interpretación de los resultados ^6^.

En Colombia, las vacunas inactivadas disponibles para la inmunización de perros con riesgo de exposición a *Leptospira* spp. contienen las serovariedades *L. canicola* y *L. icterohaemorrhagiae* (entre otras 20, aproximadamente) ^7^. No obstante, algunos estudios serológicos de leptospirosis canina en el país, mediante la prueba MAT, detectaron títulos en un rango que osciló entre 1,1 y 79,9 % ^7^, con una gran frecuencia de serovariedades diferentes a las usadas en los productos biológicos, tales como *L. fainei*, *L. tarassovi*, *L. louisiana*, *L. grippotyphosa* y *L. pomona*^7^^-^^9^.

Aunque se asume que los perros de trabajo de la Policía Nacional están bien protegidos contra la leptospirosis por estar sujetos a planes sistemáticos de inmunización, las características clínicas inespecíficas de la enfermedad (letargia, anorexia y vómito, entre otras) no son por sí solas suficientes para un diagnóstico adecuado de la enfermedad. Estas y la probabilidad de exposición a condiciones ambientales de alto riesgo, son factores que sugieren la importancia de efectuar estudios epidemiológicos que permitan conocer el estado real de la infección en los perros vacunados y la distribución espacial de la infección en ellos, para establecer si representan un problema de salud pública, en especial, para sus manejadores.

El objetivo de esta investigación fue establecer la prevalencia de *Leptospira* spp*.* en perros de trabajo vacunados y en población con riesgo ocupacional de seis unidades policiales en Colombia. Los resultados obtenidos pueden orientar las medidas preventivas apropiadas para el control de la leptospirosis en los grupos de exposición ocupacional.

## Materiales y métodos

Se llevó a cabo un estudio de corte transversal que contó con la aprobación del Comité de Ética para la Experimentación con Animales de la Universidad de Caldas (Acta No. 01 del 29 de agosto de 2017). Se obtuvo la autorización de la Policía Nacional para realizar el trabajo y el consentimiento informado previo a la toma de las muestras biológicas de humanos y perros.

### Localización geográfica

La policía cuenta con 25 unidades policiales en el territorio nacional y en el estudio se seleccionaron seis de ellas por estar situadas en lugares de clima cálido y condiciones húmedas, lo que favorece la supervivencia de *Leptospira* spp., y por los antecedentes previos de serorreacción ^10^.

Se evaluaron las unidades policiales de los municipios de Manizales (altitud de 2.170 msnm, una temperatura media anual de 17 °C y precipitación anual de 2.358 mm), Pereira (altitud de 1.411 msnm, temperatura media anual de 28 °C y precipitación anual de 2.750 mm), Armenia (altitud de 1.229 msnm, temperatura media anual de 22 °C y precipitación anual de 2.164 mm), Ibagué (altitud de 928 msnm, temperatura media anual de 24 °C y precipitación anual de 1.691 mm), Tuluá (altitud de 960 msnm, temperatura media anual de 24 °C y precipitación anual de 1.300 mm) y Cali (altitud de 985 msnm, temperatura media anual de 24 oC y precipitación anual de 1.483 mm).

### Población de estudio y tamaño de la muestra

La población de estudio en las seis remontas policiales estuvo conformada por 93 guías y 120 perros de diferentes razas (labrador retriever, golden retriever, pastor belga, pastor alemán y cruces), con edades entre 1 y 14 años, los cuales contaban con un plan previo de vacunación que incluía la inmunización sistemática con vacunas bivalentes (*L. icterohaemorraghiae* y *L. canicola*).

Se efectuó un muestreo aleatorio estratificado proporcional para la selección de las muestras con el programa Stata, versión 13.0, y se consideraron como referencia seroprevalencias nacionales de infección del 18 % en humanos (n=69) ^11^^,^^12^ y de 36,8 % en perros (n=92) ^13^. En ambos casos, se consideró un error del 5 % y un nivel de significación del 95 %.

Los criterios de inclusión de los humanos fueron tener contacto directo con los perros durante sus labores y haber estado vinculados a las unidades no menos de tres meses. Utilizando el muestreo por conveniencia, se seleccionaron dos grupos de perros, según los antecedentes de vacunación confirmada en las historias clínicas y de manejo de cada unidad policial:

a) grupo 1, perros vacunados con 11 meses de antelacion al muestreo (unidades policiales de Cali, Tulua y Armenia) y

b) grupo 2, representado por perros con inmunizacion un mes antes del muestreo (Pereira, Manizales e Ibague).

### Examen clínico

Un médico veterinario hizo el examen clínico sistemático de los perros al inicio del estudio para evidenciar síntomas relacionados con la leptospirosis ^4^^,^^14^. Esta información se consignó en un instrumento validado mediante una prueba piloto en la unidad policial de Manizales, cuya confiabilidad se determinó mediante el cálculo del alfa de Cronbach (0,75).

### Factores de riesgo

Se utilizaron dos cuestionarios estructurados y validados (alfa de Cronbach de 0,75). En el primero se evaluaron las variables demográficas, sanitarias, de manejo de los perros y sobre la presencia de especies silvestres, y se diligenció mediante visitas a las instalaciones, la observación directa y entrevistas al personal responsable. El segundo indagaba entre los participantes del estudio, su edad y su cargo, el contacto con fluidos de los perros, las normas de bioseguridad y las conductas de manejo de los animales.

### Muestras sanguíneas y prueba de microaglutinación

Se obtuvieron 5 ml de sangre por punción de la vena cefálica en humanos y perros en tubos Eppendorf sin anticoagulante, que luego se centrifugaron por cinco minutos a 500*g*; los sueros se almacenaron a 2 °C durante el transporte al laboratorio y se congelaron a -20 °C hasta el momento del análisis, efectuado 15 días después del muestreo.

Las muestras se procesaron en el Laboratorio de Diagnóstico de Agentes Biológicos, área de leptospirosis, de la Universidad del Valle en Cali. El mantenimiento de las cepas y el manejo de la técnica MAT se ajustaron a los parámetros convencionales ^13^, y se utilizaron cepas de referencia suministradas por el laboratorio internacional de referencia para el diagnóstico de la leptospirosis del *Royal Tropical Institute* (Ámsterdam, Holanda) (cuadro 1). Se consideraron como positivos los sueros de los humanos con títulos de 1:100 o más.

**Cuadro 1:** Cepas de referencia de *Leptospira* spp. suministradas por el *Royal Tropical Institute* (KIT) de Holanda usadas en la prueba MAT

Nº	Especie	Serogrupo	Serovariedad	Cepa
1	*L. interrogans*	Autumnalis	Autumnalis*	Akiyami A
2	*L. borgpetersenii*	Ballum	Ballum	Mus 127
3	*L. interrogans*	Bataviae	Bataviae	Swart
4	*L. interrogans*	Australis	Bratislava	Jez Bratislava
5	*L. interrogans*	Canicola	Canicola*	Hond Utrecht IV
6	*L. weilii*	Celledoni	Celledoni	celledoni
7	*L. kirschneri*	Cynopteri	Cynopteri	3522 C
8	*L. interrogans*	Djasiman	Djasiman	Djasiman
9	*L. kirschneri*	Grippotyphosa	Grippotyphosa*	Moskva V
10	*L. interrogans*	Sejroe	Hardjo*	Hardjoprajitno
11	*L. interrogans*	Hebdomadis	Hebdomadis*	Hebdomadis
12	*L. fainei*	Hurstbridge	Hurstbridge	BUT 6
13	*L. interrogans*	Icterohaemorrhagiae	Icterohaemorrhagiae	RGA
14	*L. inadai*	Manhao	Lichuan	Li 130
15	*L. noguchii*	Louisiana	Louisiana	LSU 1945
16	*L. borgpetersenii*	Mini	Mini	Sari
17	*L. noguchii*	Panama	Panama	CZ 214
18	*L. biflexa*	Semaranga	Patoc	Patoc I (Ames)
19	*L. interrogans*	Pomona	Pomona*	Pomona
20	*L. interrogans*	Pyrogenes	Pyrogenes	Salinem
21	*L. weilii*	Ranarum	Ranarum	ICF
22	*L. weilii*	Sarmin	Sarmin	Sarmin
23	*L. santarosai*	Shermani	Shermani	1342K
24	*L. borgpetersenii*	Tarassovi	Tarassovi	Perepelitsin

*Serovariedades que afectan y pueden mantenerse en los perros.

En el caso de los perros, se establecieron dos puntos de corte según el tiempo transcurrido entre la última vacunación y la toma de muestras sanguíneas para aumentar la especificidad, detectar verdaderas exposiciones a *Leptospira* spp. y disminuir el riesgo de incluir títulos reactivos debido a la inmunización ^15^: para el grupo 1, títulos de 1:400 o más (vacunación con 11 meses de antelación), y para el grupo 2, títulos de 1:1.600 o más (un mes de vacunados) ^2^. El grado de reacción fue interpretado al estimar los porcentajes de aglutinación de las muestras.

### Urocultivo

Las muestras de orina de los perros se obtuvieron siguiendo estrictas normas de bioseguridad; se recolectaron 15 ml de orina por medio de una sonda uretral; se filtró una alícuota de 1 ml con un filtro de 0,22 µm de nitrocelulosa marca Sartorius. Se inoculó una dilución al 10 % de la muestra de orina en medio Ellinghausen-McCullough-Johnson-Harris™ (EMJH) (Becton Dickinson and Company, Difco) ^16^.

Las muestras se mantuvieron a temperatura ambiente en recipientes térmicos protegidos de la luz solar. En el laboratorio, se incubaron a 30 °C hasta el momento en que las espiroquetas se observaron al microscopio y sin exceder en ningún caso los cuatro meses. La resiembra se hizo una vez se visualizaron las espiroquetas en el medio de cultivo de EMJH, agregando 500 µl del cultivo primario y 1 % de 5 fluorouracilo ^17^^,^^18^.

Independientemente del resultado de la lectura, se hicieron siembras para controlar la contaminación bacteriana y favorecer el crecimiento de las espiroquetas. Los cultivos se observaron con microscopía de campo oscuro (Nikon BH2); se tomaron 10 µl del cultivo y se observó toda la placa buscando la forma típica de las espiroquetas (delgadas de diferente longitud), con movimiento de traslación. La prueba se consideró positiva al observar, como mínimo, cuatro espiroquetas por placa después de 16 semanas de evaluación ^19^.

### Análisis estadístico

El análisis se efectúo utilizando el programa Stata™, versión 13.0 (College Station, Texas, USA). Se hizo un estudio descriptivo de todas las variables, se estableció la seroprevalencia de la infección en las dos poblaciones estudiadas y se analizó la regresión logística bivariada, que asumió como variable de respuesta binomial los resultados de la prueba MAT, donde 0 correspondió a los sueros con resultados negativos y 1 a los sueros positivos. Se hizo la prueba de bondad de ajuste del modelo obtenido utilizando el test estadístico de Hosmer y Lemeshow. Los efectos de las variables predictivas sobre el resultado positivo o negativo de la prueba, se expresaron mediante las razones de disparidad (*odds ratio*, OR) y sus respectivos intervalos de confianza del 95 %. Los valores de p menores de 0,05 se consideraron como significativos.

## Resultados

### Examen clínico

El 68,48 % (63/92) de los perros tenía una condición corporal normal, el 11,96 % (11/92) estaba delgado, el 15,22 % (14/92) era obeso, el 2,17 % (2/92) tenía sobrepeso y el 2,17 % (2/92) estaba caquéctico. El 11,96 % (11/92) de los animales presentaba alteraciones en el sistema tegumentario con lesiones como alopecia (27,27 %), dermatitis (18,18 %), descamación (36,36 %) y seborrea (18,18 %).

### Seroprevalencia en perros

La seroprevalencia general mediante la prueba MAT fue de 53/92 (57,61 %, IC_95%_ 0,91-2,05); se observó reacción en todas las unidades policiales evaluadas: Tuluá (80 %), Cali (60,7 %), Manizales (60 %), Pereira (53,3 %), Ibagué (42,7 %) y Armenia (40 %). La seroprevalencia de leptospirosis fue de 62,81 % (27/43) en el grupo 1 y de 53,06 % en el grupo 2 (p=0,02). El 93,48 % (n=86) de los sueros fueron reactivos con títulos de 1:100 o más y a dos o más serogrupos. Los sueros reaccionaron a todos los serogrupos evaluados con excepción de *L. autumnalis*, *L. grippotyphosa* y *L. sejroe* (cuadro 2). No se encontraron diferencias significativas entre las seroprevalencias (p=0,146) de los serogrupos. Los serogrupos predominantes (más alta titulación) fueron *L. canicola* (56,60 %), *L. mini* (11,32 %) y *L. sarmin* (7,55 %) (cuadro 3).

**Cuadro 2:** Distribución de los sueros caninos positivos para infección por leptospirosis con la prueba de microaglutinación (MAT), serovares reactivos y unidades policiales evaluadas

Serogrupos		Grupo	1		Grupo 2		Total	(%)
	Cali	Tuluá	Armenia	Manizales	Ibagué	Pereira		
*L. canicola*	12	4	1	7	4	5	33	25,78
*L. panama*	5	3	-	6	3	3	20	15,63
*L. sarmin*	4	1	-	6	5	4	20	15,63
*L. hebdomadis*	1	1	1	-	1	-	4	3,13
*L. hurstbridge*	3	-	-	-	-	-	3	2,34
*L. shermani*	1	2	1	-	1	-	5	3,91
*L. semaranga*	1	-	-	-	-	-	1	0,78
*L. cynopteri*	2	1	-	2	1	-	6	4,69
*L. tarassovi*	2	-	-	-	1	-	3	2,34
*L. djasiman*	2	3	-	-	-	1	6	4,69
*L. australis*	2	1	-	-	1	-	4	3,13
*L. mini*	5	1	-	3	-	1	10	7,81
*L. manhao*	1	-	-	-	-	2	3	2,34
*L. icterohaemorrhagiae*	-	-	-	1	-	1	2	1,56
*L. pyrogenes*	-	-	-	1	-	-	1	0,78
*L. ballum*	1	-	-	-	-	-	1	0,78
*L. louisiana*	-	1	-	-	-	-	1	0,78
*L. ranarum*	-	2	-	-	-	-	2	1,56
*L. celledoni*	1	-	-	-	-	-	1	0,78
*L. pomona*	-	-	-	-	1	-	1	0,78
*L. bataviae*	-	-	-	-	-	1	1	0,78
*L. autumnalis*	-	-	-	-	-	-	0	0,00
*L. grippotyphosa*	-	-	-	-	-	-	0	0,00
*L. sejroe*	-	-	-	-	-	-	0	0,00
Total (%)	43 33,59	20 15,63	3 2,34	26 20,31	18 14,06	18 14,06	128 100	100

**Cuadro 3:** Perros seropositivos y sus respectivas titulaciones en las seis unidades policiales evaluadas

Serogrupos	Grupo 1			Grupo 2			Total	(%)
	1:400	1:800	1:1600	1:3.200	1:1.600	1:3.200		
*L. canicola*	10	4	1	-	7	8	30	56,60
*L. panama*	-	-	-	-	-	2	2	3,77
*L. sarmin*	-	1	1	-	3	-	5	9,43
*L. shermani*	1	-	-	-	-	-	1	1,89
*L. cynopteri*	1	1	-	-	-	2	4	7,55
*L. djasiman*	1	-	-	-	-	-	1	1,89
*L. mini*	2	1	-	-	3	-	6	11,32
*L. louisiana*	-	1	-	-	-	-	1	1,89
*L. ranarum*	1	-	-	-	-	-	1	1,89
*L. celledoni*	-	1	-	-	-	-	1	1,89
*L. bataviae*	-	-	-	-	-	1	1	1,89
Total	16	9	2	0	13	13	53	100

El urocultivo fue positivo en 54 de 92 muestras (58,7 %, IC_95_ % 0,94-2,15). Se observaron espiroquetas en todas las unidades: Tuluá, 70 % (7/10); Pereira, 46,67 % (7/15); Ibagué, 50 % (7/14); Manizales, 50 % (10/20); Armenia, 20 % (1/5), y Cali, 78,57 % (22/28), con diferencias estadísticamente significativas (p=0,016).

### Seroprevalencia en humanos

La seroprevalencia de leptospirosis humana fue de 2/69 (2,89 %, IC95%:0,35-10,08): un caso en la unidad de Cali con títulos de 1:100 (*L. australis*), y otro caso en Pereira (*L. mini*) con títulos de 1:200.

### Factores de riesgo en humanos

El personal evaluado tenía contacto permanente con los perros y el 81,16 % usaba barreras de protección personal y cumplía con las normas de bioseguridad. Las dos personas que presentaron títulos contra *Leptospira* spp*.* tenían contacto con fluidos de los perros; no obstante, no se encontró ninguna asociación para la infección con las variables incluidas en el análisis (cuadro 4).

**Cuadro 4:** Frecuencia de las variables evaluadas en la población humana de las seis unidades policiales

Variable	n	(%)	p
Sexo			
Masculino	66	95,65	
Femenino	3	4,35	0,70
Edad (anos)			
24-28	9	13,04	
29-33	24	34,78	
34-38	23	33,33	
39-44	13	18,85	0,59
Cargo			
Guia canino	57	82,61	
Enfermero veterinario	8	11,59	
Servicios generales	4	5,80	0,34
Tiempo en la unidad donde labora (anos)			
1 a 6	44	63,77	
7 a 13	22	31,88	
≤14	3	4,35	0,81
Contacto con fluidos caninos			
Si	66	95,65	
No	3	4,35	0,24
Uso de medidas de bioseguridad			
Si	56	81,16	
No	13	18,84	0,87
Bano reciente en piscina			
Si	7	10,14	
No	62	89,86	0,21
Bano reciente en rios			
Si	13	18,84	
No	56	81,16	0,17
Presencia de perros en casa			
Si	39	56,52	
No	30	43,48	0,29

### Factores de riesgo en perros

Los perros mayores de diez años presentaron 6,11 veces mayores probabilidades de ser reactivos en la prueba MAT comparados con los animales de tres años o menores. En cuanto a la positividad en el urocultivo, se observaron diferencias significativas según la unidad policial evaluada. Los perros que habían consumido agua tratada presentaron menores probabilidades de resultados seropositivos a leptospiras patógenas, comparados con aquellos que habían consumido agua de nacedero *Vs*. el contacto con roedores (cuadro 5)”.

**Cuadro 5:** Análisis de regresión logística binaria de los factores asociados con la leptospirosis canina detectada mediante la técnica de microaglutinación (MAT) y urocultivo

Variables	Categoría	MAT		Urocultivo	
		OR	p	OR	p
Sexo	Macho	-	-	-	-
	Hembra	0,89	0,80	0,83	0,70
Edad (anos)	≤3	-	-	-	-
	Entre 4 y 6	1,79	0,31	0,86	0,79
	Entre 7 y 9	1,41	0,56	0,89	0,84
	≥10	6,11	0,04*	1,33	0,71
Localizacion de la unidad policial	Cali	-	-	-	-
	Tulua	2,59	0,28	0,64	0,59
	Armenia	0,43	0,40	0,68	0,03*
	Manizales	0,97	0,96	0,27	0,04*
	Ibague	0,49	0,28	0,27	0,07
	Pereira	0,74	0,64	0,24	0,04*
Acceso al agua	Roedor	-	-	-	-
por parte de otros	Gatos	1,29	0,63	0,36	0,08
animales	Perros	0,49	0,28	0,27	0,07
	Ninguno	0,74	0,64	0,24	0,04*
Acceso al alimento	Roedor	-	-	-	-
por parte de	Gatos	2,95	0,21	1,01	0,99
animales	Fauna silvestre	0,69	0,46	0,41	0,09
	Ninguno	1,11	0,86	0,43	0,15
Consumo de agua	Acueducto y nacedero	-	-	-	-
	Acueducto	1,20	0,69	0,27	0,01*
Aguas superficiales	No	-	-	-	-
	Si	1,23	0,71	1,79	0,30
Acceso a aguas	No	-	-	-	-
superficiales	Si	0,69	0,45	1,16	0,75
Pozo septico	No	-	-	-	-
	Si	1,09	0,85	2,28	0,08
Manejo de lluvias	Canalizadas	-	-	-	-
	Escorrentia y empozamiento	0,88	0,81	1,57	0,37
Registro de control	No	-	-	-	-
de roedores	Si	0,81	0,71	0,56	0,30
Senal de roedores	Heces	-	-	-	-
	Madrigueras y sendas	1,68	0,27	0,76	0,56
	Roedor muerto	0,56	0,54	0,15	0,09

* Diferencias significativas: p<0,05

- Variable de referencia

## Discusión

En el examen clínico, los perros no presentaron signos indicativos de los cuatro síndromes descritos en animales infectados por leptospiras patógenas: ictérico, hemorrágico, urémico y reproductivo ^20^; no obstante, la prevalencia general de la infección, según la prueba MAT, fue alta si se compara con los resultados de otros estudios efectuados en Colombia ^7^^-^^9^.

Es importante resaltar que el análisis y la interpretación de los resultados de MAT en perros sanos con niveles detectables de anticuerpos, son complejos y requieren cuidado para evitar errores en el diagnóstico y en la evaluación de la situación epidemiológica de la leptospirosis en población con riesgo ocupacional ^2^^,^^5^^,^^6^. Según varios autores, la interpretación depende en gran medida de cuán endémica es la enfermedad en el área de estudio, la duración de la inmunidad que confiere la vacuna ^21^^,^^22^, el lapso entre la última vacunación y la toma de las muestras sanguíneas ^6^, las limitaciones de la técnica MAT ^23^, el estado clínico de los perros evaluados ^1^ y el efecto acumulativo de las vacunaciones repetidas sobre los anticuerpos aglutinantes ^24^.

La alta seroprevalencia de leptospirosis hallada en el estudio puede estar relacionada con el riesgo ocupacional de los perros policiales evaluados. Esta actividad incluye la búsqueda de víctimas en ambientes selváticos o derrumbes o a lo largo de la orilla de los ríos, así como el contacto con suelos húmedos y cadáveres, entre otros, lo cual incrementa su riesgo de exposición natural a diferentes serogrupos de *Leptospira* spp. que son eliminados en la orina por otros reservorios domésticos o salvajes ^6^^,^^7^^,^^25^; además, representaría un desafío para su sistema inmunológico frente a infecciones asintomáticas contraídas en el campo ^1^.

Otros factores que se deben tener en cuenta son:

a) los anticuerpos aglutinantes generados por la inmunización tienen una función protectora si los epítopos o los factores antigénicos determinantes que conducen a su formación son lo suficientemente análogos a los de las cepas que infectan a los perros en la naturaleza; cuando esto no sucede, los perros pueden infectarse con otras serovariedades ^12^;

b) las vacunas son menos eficientes en la protección contra la enfermedad crónica y los portadores renales ^6^;

c) la vacunación protege al animal contra la forma aguda de la enfermedad, pero no puede prevenir la infección si los perros están expuestos a un alto riesgo infeccioso o a una cepa muy invasiva ^20^;

d) se puede presentar la enfermedad subaguda o crónica después de la infección con una cepa menos virulenta, independientemente del serogrupo vinculado ^26^, y

e) los perros que se recuperan de la infección clínica se pueden convertir en portadores renales asintomáticos ^27^.

Con relación a este último aspecto, a pesar de que el urocultivo fue positivo en una gran proporción de perros evaluados, en este estudio no se discriminó entre los serogrupos patógenos y los saprófitos, dada la baja sensibilidad y especificidad de esta prueba ^28^.

En cuanto a la duración de la inmunidad conferida por la vacuna bivalente, los resultados son discordantes, ya que la mayoría de las investigaciones se han efectuado con exposición experimental en hámsteres y cachorros jóvenes ^27^, y en pocos estudios se evalúan perros con exposición natural a las leptospiras ^2^^,^^6^^,^^25^. Estas diferencias se explican porque en los estudios experimentales se administran dosis con una gran concentración de espiroquetas por vía parenteral (instilación intraperitoneal más ocular), lo cual difiere de la exposición natural, en la cual unas pocas espiroquetas infectan al huésped mediante la penetración cutánea y mucosa (a través de la almohadilla del pie, la mucosa de la boca o la nariz), motivo por el cual la reacción inmunitaria típica cambia ^5^.

Los estudios experimentales han confirmado que la inmunización genera protección clínica a corto plazo ^24^^,^^26^^,^^29^; otros autores han demostrado que hay inmunidad a largo plazo (12 meses) ^24^^,^^27^, pero hay mucho debate sobre la protección que confiere al estado de portador renal en los perros con exposición natural ^26^^,^^27^. Según estos hallazgos, se recomienda establecer puntos de corte apropiados para analizar los resultados de la prueba MAT en perros vacunados ^25^.

En este estudio fundamentalmente epidemiológico y no clínico, en los umbrales de titulación para considerar la MAT como positiva, se tuvo en cuenta el tiempo transcurrido entre la vacunación y la toma de las muestras sanguíneas ^30^. Para el grupo de perros recién vacunados (grupo 2), se asumió como criterio positivo la presencia de títulos de 1:1.600 o más y, para los perros con 11 meses de vacunación (grupo 1), títulos de 1:400 o más, para aumentar la especificidad de la MAT y excluir la probabilidad de seleccionar sueros con títulos vacunales ^25^. Para establecer estos criterios, se tomaron como referencia los estudios efectuados por Miller, *et al*. ^25^, en perros experimentales, y por Martin, *et al.*^2^, y White, *et al.*^15^, quienes evaluaron perros infectados naturalmente. En estos casos, los perros se vigilaron serológicamente durante uno y hasta nueve años después de la vacunación; algunos perros desarrollaron títulos de 1:800 o más en las semanas 7 a 15, en tanto que en las semanas 29 a 52 ninguno de los perros tenía títulos de 1:400 o más y solo una pequeña proporción presentó títulos de 1:100 o más. Sin embargo, los autores coinciden en indicar que la cinética de anticuerpos fue variable y que, en el caso de los estudios con fines terapéuticos, sería necesario considerar otros factores que se enunciarán posteriormente.

Una vez se establece el punto de corte apropiado para la interpretación de los resultados de la MAT en los perros vacunados, se procede a identificar los serogrupos predominantes ^31^. En el presente estudio, *L. canicola* predominó en los dos grupos de perros estudiados, aspecto que puede estar relacionado con reacciones secundarias debido al esquema de vacunación, porque los títulos de 1:400 o más de este serogrupo pueden persistir durante más de un año o pueden indicar infección si el animal presenta signos sugestivos de leptospirosis ^6^, aspecto que no se observó en los perros policiales.

Los resultados evidenciaron una gran diversidad biológica, ya que se identificaron 19 de los 22 serogrupos analizados (cuadro 1). Esta biodiversidad es frecuente en las áreas tropicales o ecuatoriales y se asocia con la presencia de una amplia gama de reservorios mamíferos ^5^. No obstante, los sueros de los perros reaccionan frecuentemente a múltiples serogrupos porque las leptospiras tienen varios antígenos comunes y la MAT detecta tanto inmunoglobulinas G (IgG) como inmunoglobulinas M (IgM) ^25^^,^^32^. Sin embargo, las reacciones serológicas a otros serogrupos de *Leptospira* spp. no se pueden explicar solo por la vacunación, excepto en los casos de títulos bajos de MAT (<320), que pueden representar una aglutinación cruzada entre diferentes serogrupos ^6^. Los títulos altos, por lo general, se observan después de una enfermedad aguda con bacteriemia significativa, o como consecuencia de una infección crónica activa ^33^. Asimismo, en este estudio la alta proporción de perros con títulos ≥1:100 sugiere solo la exposición ^21^.

Los animales presentaron una mayor seroprevalencia para serovariedades que pertenecen al serogrupo *icterohaemorrhagiae* -*bratislava*, *grippotyphosa*, *pomona*, *autumnalis* y *hebdomadis-*, cuyos huéspedes de mantenimiento son los roedores ^22^. Teniendo en cuenta que los perros son los principales depredadores de muchas especies de roedores y, debido a su estrecha asociación con los humanos, constituyen un vínculo único de transmisión de roedores a humanos ^22^.

Cuando los médicos veterinarios requieran hacer un diagnóstico de leptospirosis canina en animales vacunados con fines clínicos y terapéuticos, el *American College of Veterinary Internal Medicine* (ACVIM) recomienda que se relacionen los resultados de la técnica MAT con los signos clínicos y el incremento de los títulos de MAT en dos muestras pareadas (10 a 15 días de intervalo), porque los perros con antecedentes de inmunización pueden ser positivos en la prueba, con títulos vacunales persistes de 1:1.600 o más, y con títulos de 1:800 o más no es posible confirmar un diagnóstico certero de leptospirosis ^31^.

Otros autores sugieren que, durante la primera semana de la enfermedad, el diagnóstico se efectúe con sangre evaluada mediante PCR o el aislamiento de la bacteria a partir de una muestra del paciente; después de dos semanas de evolución de la infección, este se haría a partir de los resultados de la MAT o la visualización al microscopio de campo oscuro ^5^. Sin embargo, distintos autores indican que la leptospirosis aguda se puede diagnosticar con facilidad por serología (MAT) y un aumento de los títulos (cuádruple como mínimo) en dos muestras pareadas ^21^. Por el contrario, los animales asintomáticos presentan títulos bajos y se requieren otros métodos de diagnóstico para detectar leptospiras patógenas en la orina, como la técnica PCR TaqMan modificada ^34^.

Es bien conocido que la leptospirosis canina aguda puede conducir a nefritis tubulointersticial y fibrosis intersticial que, si no se trata de manera adecuada y oportuna, puede progresar a una enfermedad renal crónica ^35^.

Asimismo, los perros que viven en las áreas endémicas se pueden infectar y mantener las leptospiras en los túbulos renales y en los intersticios por periodos prolongados, lo que conduce a una infección asintomática y a la eliminación de la bacteria en la orina ^36^.

Este mismo hallazgo se observó en los seres humanos, reportándose colonización renal por leptospiras, lo que se considera un factor de riesgo para la fibrosis renal ^35^. Por ello, se sugiere que en las zonas endémicas de leptospirosis canina en Colombia se investigue la condición de portador renal mediante reacción en cadena de la polimerasa (PCR) con el gen *LipL32* (proteína de membrana externa) en orina ^21^, la cual tiene gran sensibilidad (91,6 %) y especificidad (100 %), y permite el diagnóstico precoz de la leptospirosis clínica ^34^.

La detección temprana de portadores tiene un impacto obvio en la salud pública, y puede contribuir de manera directa en la salud animal y en la prevención de la infección renal crónica ^21^. Aunque en el presente estudio se evidenció excreción urinaria de leptospiras mediante el urocultivo, una de las limitaciones de esta técnica es que no permite la distinción entre bacterias patógenas y saprófitas, y para obtener un cultivo con crecimiento es importante la presencia y la densidad de la bacteria en la muestra biológica, lo cual depende del grado de colonización renal y de la excreción permanente, condiciones que no se cumplen en todos los casos de infección ^37^^,^^38^.

Los perros juegan un papel importante en la epidemiología de la leptospirosis humana por su estrecho contacto y su constante interacción con personas en espacios comunes, lo que aumenta la probabilidad de infección entre especies propensas ^7^^,^^39^. En estudios recientes, se ha encontrado una correlación positiva entre la leptospirosis canina y la infección humana ^40^, y en este estudio, la seroprevalencia de leptospirosis en los guías perros fue de 2,9 % y las serovariedades involucradas no están asociadas con la infección en perros (cuadro 1) ^22^. Estos resultados sugieren que las normas de bioseguridad usadas por el personal evaluado fueron efectivas ^38^, o que se requieren estudios complementarios para entender la ecología de las leptospiras patógenas en los diversos huéspedes animales, con el fin de detectar los cambios en la dinámica de transmisión ^39^.

En cuanto a los factores de riesgo asociados con la presencia de perros serorreactivos en la prueba MAT, se encontró que los de 10 años y más tuvieron 6,11 veces mayores probabilidades de tener títulos positivos que aquellos de menor edad, probablemente por tener una mayor memoria inmunológica producto de las vacunaciones repetidas y una mayor exposición natural ^41^.

Por otra parte, se observaron diferencias en los resultados del urocultivo según la localización de las unidades policiales y la presencia de fauna silvestre, lo que se explicaría por el papel de los mamíferos silvestres como huéspedes de mantenimiento de varios serogrupos de leptospiras ^42^.

Asimismo, se estableció como un factor protector para los perros, el suministro de agua potable del acueducto frente al consumo de agua directamente de nacederos sin protección ambiental ^43^, lo que favorece la supervivencia de la bacteria ^44^.

Los resultados del presente estudio evidencian que, a pesar de que los perros evaluados contaban con programas de vacunación sistemática y planes sanitarios estrictos, tenían un gran riesgo de infectarse con leptospiras patógenas durante sus actividades de trabajo. La alta seroprevalencia en los perros estudiados evidenció una presentación endémica de la enfermedad en las unidades evaluadas. El análisis de los resultados de las pruebas serológicas en perros vacunados se debe efectuar teniendo en cuenta el grado endémico de la enfermedad, el tiempo transcurrido entre la última vacunación y la toma de las muestras sanguíneas, la definición de puntos de corte apropiados, el análisis de los títulos prevalentes de los serogrupos evaluados por MAT, los anamnésicos y la presencia de signos clínicos, entre otros aspectos. También, es urgente conocer los diferentes algoritmos diagnósticos para diferenciar los anticuerpos IgM e IgG mediante técnicas alternativas como la inmunofluorescencia indirecta (IFI). En el caso de estudios con fines clínicos y terapéuticos, se sugiere tomar muestras pareadas para el análisis serológico con la MAT, y el uso de técnicas moleculares sensibles y específicas.
